# Effectiveness of psychotherapy in improving medication adherence for hypertension and diabetes mellitus among displaced Myanmar patients in Thailand: A randomized control trial

**DOI:** 10.1017/gmh.2026.10225

**Published:** 2026-05-26

**Authors:** Wongsa Laoasiriwong, Ye Htut Oo, Kittipong Sornlorm, Judith Bass, Stephanie Van Wyk Skavenski, Laura Murray, Amanda Nguyen, Kaung Nyein Aye, Jarntrah Sappayabanphot, Srishti Sardana, Catherine Lee, Ashley Leichner

**Affiliations:** 1 Faculty of Public Health, Khon Kaen University, Thailand; 2 Johns Hopkins University Bloomberg, School of Public Health, USA; 3 Department of Mental Health, Johns Hopkins Bloomberg School of Public Health, USA; 4 School of Education & Human Development, University of Virginia, USA; 5 International Rescue Committee, USA; 6 International Rescue Committee, Thailand; 7 Department of Psychology, University of Wisconsin System, USA

**Keywords:** psychotherapy, diabetes, displaced population, hypertension, medication adherence

## Abstract

Displaced persons with non-communicable diseases (NCD) face challenges in disease management and increased risk of mental health problems. This randomized controlled trial assessed the impact of psychotherapy treatment (Common Elements Treatment Approach [CETA]) on hypertension and diabetes mellitus medication adherence (primary) and mental and physical health outcomes (secondary) among displaced Myanmar adults with poor medication adherence in a Thai shelter community. Treatment participants received weekly CETA for 4–12 weeks; comparison participants had access to routine psychosocial support. Eligible participants were chronic disease patients with <70% medication adherence over prior month. Data were collected at baseline, 3 and 6 months. Of 650 adults screened, 283 (43.5%) met eligibility criteria and 224 consented (CETA n = 112, comparison n = 112) to participate; 73 (32.6%) reported elevated mental health symptoms. At 6-month follow-up, medication adherence improved in both arms, with greater improvements among CETA participants (MARS-5: 0.55, 95% CI: 0.07 to 1.02, p = 0.023). CETA significantly reduced mental health symptoms among those with elevated symptoms at baseline (−3.06, 95% CI: −5.19 to −0.93, p = 0.005). The high rate of mental health symptoms and the impact of CETA on NCD medication adherence supports integration of mental health services into NCD care systems.

## Impact statement

This study provides novel evidence that a scalable mental health intervention can improve medication adherence for chronic diseases among displaced populations living in humanitarian settings. Non-communicable diseases (NCDs) such as hypertension and diabetes are increasingly prevalent in refugee contexts, yet adherence to long-term treatment remains poor due to psychological distress, structural barriers and limited health system capacity. By demonstrating that the Common Elements Treatment Approach (CETA) improves self-reported medication adherence, as well as mental health outcomes for those suffering from elevated symptoms, among displaced Myanmar adults in Thailand, this trial highlights the value of integrating mental health services into routine NCD care. The findings are particularly relevant for humanitarian health programming, as they show that transdiagnostic psychotherapy can address behavioral health outcomes beyond traditional mental health symptoms, even among individuals without elevated psychological distress. This expands the potential role of mental health interventions in strengthening chronic disease management in low-resource and crisis-affected settings. For policymakers, practitioners and humanitarian organizations, this study supports investment in integrated care models that combine mental health and chronic disease services. Such approaches may enhance treatment adherence, improve quality of life and reduce preventable complications from NCDs among displaced and underserved populations globally.

## Introduction

Non-communicable diseases (NCDs) are the primary cause of death globally, with 77% of these deaths occurring in low- and middle-income countries (LMICs) (World Health Organization, 2023). The burden of NCDs is on the rise among people living in humanitarian settings in LMICs due to displacement, disrupted healthcare systems and inaccessibility to services (Aebischer Perone et al., 2017; Ngaruiya et al., 2022). Displaced persons with chronic diseases such as hypertension (HT) and diabetes mellitus (DM) face numerous context-related difficulties in managing their disease conditions, such as poor living conditions and limited access to healthy food, safe environments and consistent healthcare (International Organization for Migration, 2018). These populations are also more susceptible to develop mental health problems due to numerous physical and psychological stressors (Papola et al., 2020).

Since 1980s, people from Myanmar have been fleeing to neighboring countries due to the prolonged political instability. Thailand hosts temporary shelters along its border with Myanmar, and the displaced populations from Myanmar have been entering the temporary shelters for the last 40 years (Soe and Worland, 2024). There are multiple shelters in the region run by the Royal Thai Government with Mae La the largest among them, with 21,224 adults living there as of June 2019 (The Border Consortium, 2021). Residents of these shelters face a myriad of vulnerabilities, including limited work opportunities, resource constraints and ongoing stress. While healthcare access is limited, the International Committee (IRC) does provide medical and psychosocial supports to people living Mae La and according to their NCD clinical database (International Rescue Committee, 2019), there were 6,793 HT cases per 100,000 population and 2,409 DM cases per 100,000 population in the Mae La shelter in 2019.

Managing chronic conditions such as HT and DM often includes adherence to prescribed medications and lifestyle behavioral modifications (Abu et al., 2018; Agidew et al., 2021). A focus on medication adherence is motivated in part because poor- or non-compliance can compromise an individual’s health and can contribute to the emergence of complications and increased healthcare costs (World Health Organization, 2003). A scoping review by Chauke et al. (2022) of factors influencing poor medication adherence among patients with chronic disease in LMIC identified patient beliefs, knowledge and attitudes as key factors within the patient’s control. This fits with the social-behavioral frameworks described by Amico et al. (2018) that acknowledge the complexity of factors that go into understanding and addressing human behavior, including the self-administration of medication. Amico and colleagues note that adherence behavior change includes factors such as motivation, coping, affect regulation and decision making.

There are a range of different approaches that have been tested to improve medication adherence, including patient incentives and education, medication regimen management and reminders and cognitive behavioral therapies. In their review, Kini and Ho (2018) report that the cognitive behavioral therapies most frequently used to improve adherence include motivational interviewing, planned behavior education and self-management strategies, with the most successful interventions delivered by trained counselors over multiple sessions. A systematic review and meta-analysis of interventions to improve medication adherence in hypertensive patients found that the most promising elements included patient feedback, self-monitoring of blood pressure, using pill boxes and other special packaging and motivational interviewing, with the most successful interventions using multiple elements delivered over multiple sessions (Conn et al., 2015).

In addition to the multiple factors associated with poor medication adherence, the link between poor treatment compliance and mental health is well documented in the context of HT and DM, with the relationship between mental health and NCDs being bidirectional in associations (DiMatteo et al., 2000; Gonzalez et al., 2008; Robinson et al., 2018; Herrera et al., 2021). Addressing mental health concerns and behavioral factors associated with poor NCD medication adherence could have a synergistic effect to improve both types of conditions and quality of life.

Results from prior qualitative data collection conducted with Mae La-based NCD patients, caregivers and healthcare workers identified mental health counseling and psychosocial supports as potentially useful to manage mental health problems such as depression and anxiety as well as frustrations and challenges with managing medication compliance and dietary changes (International Rescue Committee, 2019). Global research has also found that psychosocial interventions can improve health outcomes for patients with diabetes (Pascoe et al., 2017; Massey et al., 2019). Yet, there is a gap in our understanding of whether interventions that have been developed and tested to improve mental health outcomes can also address the motivational and behavioral factors associated with poor medication adherence in the absence of mental health concerns, particularly among people living in humanitarian settings with few resources and complex trauma experiences.

The Common Elements Treatment Approach (CETA) is an evidence-based transdiagnostic intervention that is designed to address multiple mental and behavioral health problems through personalized treatment based on the presenting concerns of each client (Murray et al., 2014). CETA counselors are trained in distinct evidence-based elements, such as psychoeducation, behavioral activation, cognitive coping, anxiety management, exposure and motivational interviewing, which they deliver in varying doses, combinations and sequences based on the patient’s presenting problems and needs. Supervisors support the CETA counselors in element selection, dosing and order of delivery. A previous trial with displaced Myanmar adults who reported trauma exposure and elevated symptoms of depression and/or posttraumatic stress showed effectiveness for reducing depression, anxiety and posttraumatic stress symptoms and improving facets of daily functioning (Bolton et al., 2014). Trial participants received between 7–13 CETA sessions. All participants received the psychoeducation, cognitive coping, imaginal gradual exposure and safety elements, with variations provided for the behavioral activation, substance use and anxiety management elements.

In this study, we aimed to evaluate the impact of the CETA model on medication adherence among adults with poor adherence to their HT and/or diabetes mellitus (DM) medications with and without comorbid mental health problems. We also explored the impact of CETA participation on mental health and HT- and DM-specific clinical outcomes.

## Methods

### Study design

The study used a two-arm parallel group randomized controlled trial, comparing CETA (n = 112) to access to existing available mental health and psychosocial (MHPSS) services (n = 112). The study took place in Mae La shelter, which was established in 1984 in Tha Song Yang District of Tak Province in Thailand on the border with Myanmar. The shelter community is set up with 3 main zones and 22 sections. IRC has a primary healthcare facility in each zone with the NCD clinic rotating operations on different days in each zone. Participants randomized to the CETA program could choose to receive their weekly counseling in a private room at a primary healthcare facility, at the main IRC office in Zone C, or at their homes. The comparison group could receive mental health services through the MHPSS center in each zone, with follow-up services also available at their home. Ethics approval was obtained from the Center for Ethics in Human Research at Khon Kaen University, Thailand, under the reference number of HE652103; the trial was registered on ClinicalTrials.gov (NCT05512624).

### Participants

The study population was displaced adults from Myanmar with HT and/or type-2 DM who had poor medication adherence, defined as taking less than 70% of the prescribed medications over the past month. A list of adult HT and DM patients (n = 1,712) was obtained from the IRC chronic disease care system database. Study data collectors were given lists of 10 adults at a time in a particular zone and went to their homes to inform them of the study and complete an oral consent to screen for study eligibility. Once consented, the data collector conducted a pill count of all available DM and/or HT medications; patients were identified as study eligible if the observed pill count for any DM or HT medication was <70% of what was expected. Study exclusion criteria included being younger than 18 years of age, having a severe mental or physical illness that limited their ability to participate in a weekly therapy program and being unable to communicate verbally. Pill count data and basic demographic information were recorded by the data collector on a tablet.

**Figure 1.**
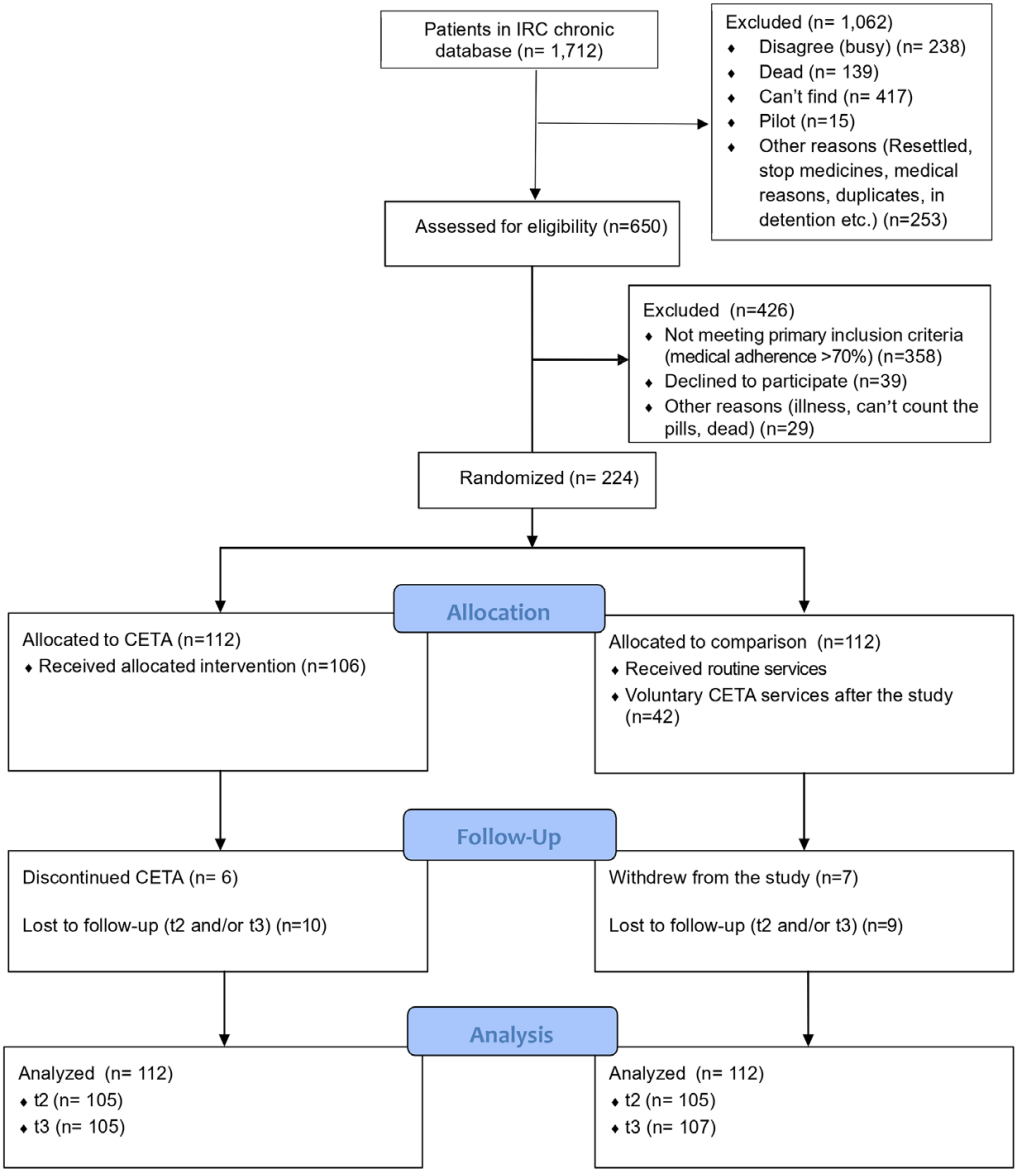
Trial Profile. Figure 1. long description.

Participants who met eligibility criteria were provided with more study information and a written trial consent; those who consented to participate completed a full baseline assessment, which included demographics, self-reported medication adherence, mental health and other clinical outcomes. Participants were followed up at 3 and 6 months with the same full assessment. Over the course of the trial, we maintained contact with 102 (91%) of the CETA treatment participants and 103 (92%) of the comparison participants (Figure 1).

### Randomization and masking

At time of trial consent, study participants were allocated to treatment conditions using a stratified randomization schema, with stratification based on NCD disease (HT, DM, HT + DM) generated by the study research manager. The study research manager (YHO) put the treatment condition information on a small piece of paper, which was folded and stapled to the back of each pre-numbered consent form. After receiving written trial consent and collecting the baseline assessment, data collectors revealed the condition assignment to the participants by unsealing the treatment condition paper. Each data collector was provided with a certain number of consent forms, which were tracked to ensure they were used in numerical sequence. Participants completed the full assessment at the baseline (t1), 3 months after the baseline (after completing the CETA program for those in the treatment arm) (t2), and 6 months after the baseline (t3). Data collectors were blinded to the assigned treatment condition of study participants at follow-up assessments.

### Intervention procedures

All study participants received routine non-communicable disease (NCD) services, including medications and lifestyle counseling, provided by IRC staff and camp-based assistants in Mae La.The NCD protocol was based on the Burmese Border Guideline 2020 (Shoklo Malaria Research Unit, 2020).

The CETA (Murray et al., 2014) is a flexible, modular, transdiagnostic intervention that was implemented on an individual basis for this study. CETA providers, who were Mae La residents, were trained to select treatment elements based on the patient’s presenting symptoms and problems as assessed using mental health outcomes measures (see secondary outcomes below). The number of sessions ranged from 4–12 weekly 1-h sessions, with fewer sessions for patients with poor adherence but no mental health concerns and more sessions for patients with poor adherence and comorbid mental and/or behavioral health problems. CETA counselors were supervised weekly by local IRC supervisors who were supervised by CETA trainers based in Yangon, Myanmar, and overseen by the CETA development team (SS). The CETA trainers and local supervisors reviewed the initial CETA treatment plan for each study participant, including the selection of elements, dosing and order of delivery. During the weekly supervision, the local supervisors reviewed the elements completed, any challenges faced and plans for the subsequent session. In general, each provider delivered CETA to 7–12 participants over the course of the study, with the exception of two providers who left the project temporarily and rejoined before the end of the study.

Participants in the comparison condition were reminded of availability of the currently available MHPSS services available at the MHPSS center at each zone; these services included a range of programs, including psychoeducation to clients and families, relaxation activities, individual counseling and referral to district hospitals for specialized psychiatric care and follow-up home visits. As standard practice, the MPHSS programs were not formalized such that each person who came to an MHPSS center was informed of the different available services and in conversation with center staff would decide what they wanted to participate in. The MHPSS center services were delivered by camp-based psychosocial workers who had received training in the IRC Thailand’s MHPSS curriculum and were supervised by a psychosocial manager and officer from IRC Mae Sot Office. We did not conduct formal tracking of uptake of MHPSS services by study participants.

### Outcomes

The primary outcome of medication adherence was measured in two ways: pill count identification conducted by a study data collector and self-reported 5-item Medication Adherence Rating Scale (MARS-5) (Chan et al., 2020). Pill count was calculated by subtracting the number of remaining medications from the number of medications originally dispensed and divided by the expected number to be taken. It was expressed as a percentage by multiplying the result by 100 (the percentage of the pill count was automatically calculated by the application to limit individual error). Dispensary information was calculated by review of the patient’s medication book, which included information from the pharmacy. Data collection was monitored on a weekly basis by the research manager through review of data and meetings with data collectors. Pill count data were classified into four categories: good adherence (≥70%), moderate adherence (60–69%), low adherence (50–59%) and very low adherence (<50%). The MARS-5 scale is comprised of 5 items regarding the patients’ noncompliance behavior to medications during the past 2 weeks: *forget to take medication(s), change the dosage, stop taking them for a while, skip one of the dosages and use less than it is prescribed.* It has 5-point Likert Scale (0 = never, 1 = a few days, 2 = about half the days, 3 = most days, 4 = all day). During the analysis, the score was reversed and started with 1 for always to 5 for never. Higher scores indicated higher medication adherence reported by the participants.

Secondary outcomes included self-reported mental health symptoms and HT and DM-specific clinical outcomes (e.g. disease type, blood pressure and fasting blood sugar [FBS] using HbA1c). Mental health symptoms, focused on depression, anxiety and posttraumatic stress symptoms, were measured using a 15-item tool based on the International Depression Symptom Scale (IDSS) and the Applied Mental Health Research (AMHR) group short instrument (Applied Mental Health Research Group, 2017; Haroz et al., 2017). These conditions were selected in consultation with the local MHPSS providers who regularly see patients with these symptoms. Participants were asked about the frequency of how often they experienced each symptom within the prior 2 weeks, with the response options ranging from 0, “not at all,” to 3, “almost all of the time” with a total score ranging from 0 to 45. The tool was previously validated in both Myanmar language and S’gaw Karen. A total score of 6 or above was defined as the cut-off for elevated mental health symptoms. The study also assessed substance use, eating and exercise behaviors, which are not presented in this paper as they were considered exploratory outcomes.

Information on NCD type (HT and/or DM) was extracted from the IRC chronic disease database system. Blood pressure was monitored by data collectors at all assessments. Blood for HbA1C testing was collected from participants with DM by nurses at baseline (t1) and final (t3) assessments and sent to a district hospital for analysis. To define high blood pressure, the cutoff for systolic blood pressure (SBP) was 140 mmHg, and the cutoff for diastolic blood pressure (DBP) was 90 mmHg (Martin, 2008). To define high blood sugar, the cutoff score for FBS level was ≥126 mg/dL (American Diabetes Association, 2021) and ≥7% for HbA1C (Baranwal et al., 2020).

Suicide and homicide ideation safety assessments and gender-based violence (GBV) screening occurred at each assessment (t1, t2 and t3) by data collectors and during each counseling session by CETA providers. Referrals for high-risk participants who reported any ideation and/or GBV were made to appropriate care authorities. Other medical conditions were checked during routine follow-up visits to IRC clinics by medics and doctors as needed.

### Statistical analysis

The sample size for this randomized controlled trial was calculated using G*Power software (Faul et al., 2007). A priori power analysis was performed for a two-tailed test comparing the means of two independent groups, with an effect size (Cohen’s d) of 0.5, an alpha error probability (α) of 0.05 and a power (1 – β) of 0.95 (Cohen, 2013). The analysis assumed an equal allocation ratio of (1:1) between the intervention and comparison groups. The results indicated that a total of 210 participants were required, with 105 participants in each group, to ensure adequate power to detect a medium effect size with statistical significance.

Data analysis was intention-to-treat; the full sample was used for the primary medication adherence outcomes, while secondary outcome analyses (i.e. mental health symptoms and clinical outcomes) were restricted to participants with these problems at baseline. We used analysis of covariance and generalized estimating equations (GEE) for primary and secondary outcomes, using Stata version 18.0 (College Station, Texas 77845 USA). All outcomes were analyzed as continuous. Effect sizes were calculated for the primary medication adherence outcomes using Cohen’s d, which was computed by taking the difference in mean scores between the CETA and comparison groups, and dividing by the pooled standard deviation (SD) at each follow-up (midline and endline). No data monitoring committee was established for this study as the intervention was determined to pose no additional risk to participants and appropriate protocols were already in place for safety monitoring.

## Role of the funding source

The funder was the Research for Health in Humanitarian Crises (R2HC) program of Enhancing Learning and Research for Humanitarian Assistance. The funder of the study had no role in study design, data collection, data analysis, data interpretation or writing of the report.

## Results

Over the course of study recruitment (September 2022 to September 2023), 1,712 patients were identified in the IRC’s chronic disease database. We screened 650 (37.97%) NCD patients for eligibility, with 283 (43.5%) meeting adherence eligibility criteria and 224 (79% of eligible) consenting to participate. Half (n = 112) were randomized to the CETA treatment and half (n = 112) to the comparison condition. Over the course of the trial (last data collection February 2024), we maintained contact with 102 (91%) of the CETA treatment participants and 103 (92%) of the comparison participants (Figure 1).

The participant characteristics were similar across treatment arms (Table 1). Continuous variables were compared using independent samples *t*-tests, while categorical variables were compared using chi-square tests or Fisher’s exact tests as appropriate. The mean age in the intervention group was 56.08 years (SD: 12.86) while in the comparison group, it was 55.27 years (SD: 11.68). In terms of sex distribution, 32.14% of the intervention group were male compared to 25.89% in the comparison group, with females making up the majority in both (67.86% and 74.11%, respectively). The ethnic composition was similar across conditions with Karen participants comprising 70.54% of both groups. Ethnicities such as Bamar (8.93% vs. 2.68%), Kachin, Mon and others made up the remaining participants.

**Table 1.** Baseline characteristics of the participants (n = 224) Table 1. long description.

Factors	Intervention (n = 112)		Comparison (n = 112)		p-value
	No.	%	No.	%	
Age					0.621^a^
Mean (SD)	56.08 (12.86)		55.27 (11.68)		
Median (Min: Max)	57.5 (24: 88)		56 (26: 88)		
Sex					0.303^b^
Male	36	32.14	29	25.89	
Female	76	67.86	83	74.11	
Marital status					0.088^b^
Single, separated, divorced	43	38.39	31	27.68	
Married	69	61.61	81	72.32	
Ethnic group					0.231^c^
Karen	79	70.54	79	70.54	
Bamar	10	8.93	3	2.68	
Kachin	2	1.79	1	0.89	
Mon	2	1.79	3	2.68	
Others	19	16.96	26	23.21	
Religion					0.369^b^
Christianity	31	27.68	34	30.36	
Buddhism	46	41.07	36	32.14	
Islam	35	31.25	42	37.50	
Duration of stay at the shelter (years)					0.348^b^
<10	6	5.36	7	6.25	
10–19	67	59.82	76	67.86	
≥20	39	34.82	29	25.89	
Mean (SD)	18.32 (6.87)		16.58 (5.95)		
Median (Min: Max)	17 (1: 35)		16 (1: 32)		
Number of household members					0.708^b^
1–3	26	23.21	21	18.75	
4–6	54	48.21	58	51.79	
>6	32	28.57	33	29.46	
Category of disease					0.741^b^
Hypertension	69	61.61	74	66.07	
Diabetes	22	19.64	18	16.07	
DM and HT comorbidity	21	18.75	20	17.86	

*Note:* p-value of ^a^t-test, ^b^Chi-square test and ^c^Fisher’s exact test.

Duration of stay at the shelter was categorized as <10 years, 10–19 years and ≥ 20 years and was comparable between groups. The mean duration of stay at the shelter was slightly longer in the intervention arm (18.32 years, SD: 6.87) compared to the comparison (16.58 years, SD: 5.95). Household sizes were comparable with 48.21% in the intervention arm and 51.79% in the comparison, reporting 4 to 6 household members.

NCD health conditions were comparable across arms, with HT being the most common NCD (61.61% in the intervention arm and 66.07% in the comparison). DM alone affected 19.64% of the intervention arm and 16.07% of the comparison, while comorbidity of HT and DM was present in 18.75% and 17.86%, respectively. No statistically significant differences were observed between the two arms for any baseline characteristic (p > 0.05 for all comparisons).

## Medication adherence: Pill count identification and self-reported 5-item Medication Adherence Rating Scale (MARS-5)

The first part of the Table 2 presents the primary outcome of medication adherence by treatment condition and assessment time. At baseline, while all study participants had poor medication adherence based on pill count (defined as <70%), with the majority met criteria for very low adherence (<50%) with 75% of the CETA group and 79.46% of the comparison group falling into this category. At t2, adherence significantly improved in both groups and by 6-month follow-up (t3), 72.38% of the intervention participants and 68.22% of the comparison moved into the “good adherence” classification (>70%) with very low adherence decreasing to 20% in both groups. Mean pill adherence increased substantially from baseline to t3 follow-up. The CETA group showed a large and significant pill count improvement from t1 to t2 (mean difference = 54.06, 95% CI: 45.79 to 62.32, p < 0.001), which was maintained at t3 (mean improvement = 54.64, 95% CI: 46.37 to 62.90, p < 0.001). The comparison group also demonstrated significant pill count improvements from t1 to t2, with a mean improvement of 48.73 (95% CI: 39.94 to 57.53, p < 0.001), which was maintained at t3 (mean improvement = 47.73 (95% CI: 38.97 to 56.48, p < 0.001).

**Table 2.** Medication adherence among participants with hypertension and/or diabetes mellitus at each assessment time and the secondary outcomes (mental health symptoms, blood pressure and blood sugar levels) among participants who met baseline criteria for the different outcomes (mental health symptoms, hypertension elevated SBP and DBP, and/or diabetes with elevated fasting blood sugar) Table 2. long description.

Factors	t1: Baseline				t2: 3-month				t3: 6-month			
	CETA		Comparison		CETA		Comparison		CETA		Comparison	
	No.	%	No.	%	No.	%	No.	%	No.	%	No.	%
Pill count	N = 112		N = 112		N = 105		N = 105		N = 105		N = 107	
Good adherence (≥70%)					73	69.52	65	61.90	76	72.38	73	68.22
Moderate adherence (60–69%)	14	12.50	15	13.39	6	5.71	10	9.52	6	5.71	6	5.61
Low adherence (50–59%)	14	12.50	8	7.14	6	5.71	6	5.71	2	1.9	2	1.87
Very low adherence (<50%)	84	75.00	89	79.46	20	19.05	24	22.86	21	20.0	26	24.3
Mean (SD)	22.41 (24.72)		23.27 (24.33)		76.47 (34.18)		72.01 (34.70)		77.05 (33.34)		71.00 (38.46)	
Mean difference (95% CI) from baseline assessment	*Na*		*Na*		54.06 (45.79 to 62.32)***		48.73 (39.94 to 57.53)***		54.64 (46.37 to 62.90)***		47.73 (38.97 to 56.48)***	
					Effect size: 0.13 (95% CI: – 0.14 – 0.40)				Effect size: 0.17 (95% CI: −0.10 – 0.44)			
MARS–5 in the past 2 weeks	N = 112		N = 112		N = 105		N = 105		N = 105		N = 107	
Poor (≤7)	2	1.79	0	0	0	0	0	0	0	0	0	0
Fair (8–15)	5	4.46	7	6.25	2	1.90	1	0.95	1	0.95	2	1.87
Adequate (16–25)	105	93.75	105	93.75	103	98.10	104	99.05	104	99.05	105	98.13
Mean (SD)	20.83 (3.57)		20.91 (3.23)		23.94 (2.16)		23.11 (2.25)		24.14 (1.68)		23.64 (2.53)	
Mean difference (95% CI) from baseline assessment	*Na*		*Na*		3.11 (2.41 to 3.81)***		2.28 (1.56 to 3.01)***		3.31 (2.61 to 4.01)***		2.72 (2.00 to 3.44)***	
					Effect size: 0.38 (95% CI: 0.10–0.65)				Effect size: 0.23 (95% CI: −0.04 – 0.50)			
Mental health symptoms	N = 36		N = 37		N = 36		N = 37		N = 36		N = 37	
Low (score: 0–5)		–	–	–	30	83.33	18	48.65	26	72.22	23	62.16
Elevated (score: ≥6)	36	100	37	100	6	16.67	19	51.35	10	27.78	14	37.84
Mean (SD)	13.02 (7.35)		11.67 (6.52)		3.50 (3.41)		6.70 (5.42)		3.83 (4.97)		6.00 (8.18)	
Mean difference (95% CI) from baseline assessment					−9.52 (−11.87 to −7.18)***		−4.97 (−7.13 to −2.81)***		−9.19 (−11.67 to −6.70)***		−5.67 (−8.55 to −2.79)***	
Blood pressure (SBP ≥ 140; DBP ≥ 90)	N = 43		N = 47		N = 43		N = 47		N = 43		N = 47	
Normal	–	–	–	–	18	41.86	22	46.81	30	69.77	27	57.45
High	43	100	47	100	25	58.14	25	53.19	13	30.23	20	42.55
Systolic: mean (SD)	141.81 (16.06)		138.40 (18.37)		131.88 (21.27)		131.31 (18.14)		124.41 (19.08)		125.95 (21.04)	
Mean difference (95% CI) from baseline assessment			*-*		−9.93 (−16.20 to −3.65)**		−7.08 (−13.48 to −0.68)*		−17.39 (−24.51 to −10.27)***		−12.44 (−18.75 to −6.13)***	
Diastolic: mean (SD)	91.09 (8.11)		93.80 (15.30)		84.74 (18.59)		82.21 (11.66)		80.04 (11.06)		79.72 (10.26)	
Mean difference (95% CI) from baseline assessment			*-*		−6.34 (−11.88 to −0.81)*		−11.59 (−16.21 to −6.97)***		−11.04 (−14.53 to −7.55)***		−14.08 (−19.00 to −9.16)***	
Fasting Blood Sugar (≥126 mg/dL)	N = 30		N = 24		N = 30		N = 24		N = 30		N = 24	
Normal	–	–	–	–	3	10.00	3	12.50	7	22.58	4	17.39
High blood sugar	30	100	24	100	27	90.00	21	87.50	24	77.42	19	82.61
Mean (SD)	205.30 (58.63)		176.41 (40.40)		195.13 (66.07)		196.66 (76.83)		184.00 (67.58)		168.91 (49.47)	
Mean difference (95% CI) from baseline assessment					−10.16 (−7.30 to 27.64)		−20.25 (−59.04 to 18.54)		−25.43 (−49.79 to −1.07) *		−7.47 (−32.15 to 17.20)	
HbA1C (≥7%)	N = 28		N = 29						N = 28		N = 29	
Normal	–	–	–	–					2	3.57	2	6.90
High blood sugar	28	100	29	100					26	96.43	27	93.10
Mean (SD)	9.55 (1.94)		8.72 (1.35)						9.35 (2.04)		8.70 (1.56)	
Mean difference (95% CI) from baseline assessment									−0.31 (−0.90 to 0.28)		−0.02 (−0.70 to 0.66)	

*Note:* *: p-value < 0.05, **: p-value < 0.01, ***: p-value < 0.001.

For self-reported adherence measured using the MARS-5 scale, the baseline assessment indicated that both groups had adequate self-reported adherence (93.75% of the intervention group and 93.75% of the comparison group had scores in the adequate range of 16–25). At follow-up assessments, the percentage of participants with adequate self-reported adherence remained high in both groups. For MARS-5 mean scores, the CETA group showed large and significant improvements from t1 to t2 (mean difference = 3.11, 95% CI: 2.41 to 3.81, p < 0.001), which were maintained at t3 (mean difference = 3.31, 95% CI: 2.61 to 4.01, p < 0.001). The comparison group also showed significant improvements, with a mean improvement of 2.28 at t2 (95% CI: 1.56 to 3.01, p < 0.001), which was maintained at t3.

Table 3 presents the comparison of medication adherence improvements by treatment condition. At t2, the CETA group showed a significantly higher mean improvement in medication adherence compared to the comparison group for the self-reported MARS-5 measure and large, but not statistically significant, difference on the mean pill count outcome. This trend was maintained for the overall difference across both follow-up periods (t2 to t3). The overall difference was significant, with a mean difference of 0.55 (95% CI: 0.07 to 1.02, p = 0.023), indicating that CETA had a sustained impact on medication adherence when measured by the MARS-5 scale.

**Table 3.** Adjusted post-intervention difference and overall difference among 2 follow-ups (t2 and t3) between comparison and CETA groups for medication adherence Table 3. long description.

Primary healthcare, health status and improving medication adherence factors	Adjusted* post-intervention difference between CETA and comparison (ref) by ANCOVA				Overall difference between CETA and comparison (ref) among 2 follow-up assessments (t2-t3) by GEE	
	t2		t3			
	Mean difference (95% CI)	p-value	Mean difference (95% CI)	p-value	Mean difference (95% CI)	p-value
**Medication nonadherence**						
Pill counts	4.77 (−4.54 to 14.08)	0.314	5.71 (−4.03 to 15.46)	0.250	4.96 (−2.26 to 12.19)	0.178
MARS–5	0.73 (0.14 to 1.33)	0.015	0.39 (−0.12 to 0.91)	0.137	0.55 (0.07 to 1.02)	0.023

* Adjusted: Baseline.

### Mental health and clinical outcomes (disease type, blood pressure and blood sugar levels: Fasting blood sugar, HbA1c)

Study participants (n = 73) had elevated mental health symptoms at baseline (n = 36 in CETA; n = 37 in comparison). Participants in CETA showed a notable reduction in mean scores from 13.02 (SD = 7.35) at baseline to 3.83 (SD = 4.97) at 6-month (t3) follow-up, while the comparison group showed a smaller reduction from 11.67 (SD = 6.52) at baseline to 6.00 (SD = 8.18) at 6-months. Regarding the within-group changes in mental health symptoms, the CETA participants showed greater improvement compared to the comparison participants. The former showed a statistically significant improvement with a mean difference of −9.19 (−11.67 to −6.70), while the latter showed a mean difference of −5.67 (−8.55 to −2.79) at the overall follow-up (t3 compared to t1). The results of the overall adjusted difference between the CETA and comparison groups (midline-endline) based on GEE in Table 4 showed that the CETA participants exhibited a greater reduction in mental health symptoms compared to the comparison participants (mean difference = −3.06, 95% CI: −5.19 to −0.93, p = 0.005) (Table 4).

**Table 4.** Adjusted post-intervention difference and overall difference among 2 follow-up assessments (Midline-Endline) between comparison and CETA groups for secondary outcomes among affected sample at baseline Table 4. long description.

Primary healthcare, health status and improving medication adherence factors	Adjusted* post-intervention difference between comparison and CETA groups by ANCOVA				Overall difference between comparison and CETA groups among 2 follow-up assessments (midline-endline) by GEE	
	Midline		Endline			
	Mean difference (95%CI)	p-value	Mean difference (95%CI)	p-value	Mean difference (95%CI)	p-value
**Primary healthcare**						
Mental health symptoms	−3.53 (−5.52 to −1.55)	0.001	−2.58 (−5.63 to 0.45)	0.094	−3.06 (−5.19 to −0.93)	0.005
Hypertension						
Systolic blood pressure	−0.80 (−8.61 to 7.00)	0.838	−2.72 (−10.86 to 5.42)	0.509	−1.76 (−8.02 to 4.49)	0.581
Diastolic blood pressure	3.46 (−2.79 to 9.73)	0.274	0.84 (−3.56 to 5.25)	0.704	2.15 (−1.87 to 6.19)	0.295
Fasting blood plasma	−16.47 (−54.50 to 21.54)	0.388	−1.38 (−33.06 to 30.28)	0.930	−9.05 (−34.06 to 15.95)	0.478
HbA1C	Na	Na	0.59 (0.34 to 0.84)	<0.001	Na	Na

* Adjusted: Baseline.

Among the n = 90 study participants with elevated BP (n = 43 in CETA arm; n = 47 in comparison arm), CETA participants demonstrated a significant improvement, with a decrease in mean SBP from 141.81 (SD = 16.06) at baseline to 124.41 (SD = 19.08) at 6-month follow-up (t3). The comparison group showed a smaller decrease from 138.40 (SD = 18.37) at baseline to 125.95 (SD = 21.04) at t3. CETA participants showed more improvement than those in the comparison condition, with a mean difference of −17.39 (−24.51 to −10.27) versus −12.44 (−18.75 to −6.13). In terms of DBP, participants in both conditions experienced reductions, but the comparison participants showed a more significant decrease from 93.80 (15.30) at baseline to 79.72 (10.26) at 6-month follow-up (t3) compared to the CETA group, which decreased from 91.09 (8.11) at baseline to 80.04 (11.06) at 6-months. In terms of within-group changes, the comparison group showed a statistically significant improvement with a mean difference of −14.08 (−19.00 to −9.16), while the CETA group showed a mean difference of −11.04 (−14.53 to −7.55) at the overall follow-up (t3 compared to t1).

Fifty-four participants had baseline elevated FBS (n = 30 in CETA; n = 24 in comparison); with levels dropping for both groups over the course of the study, with the CETA group showing a reduction in mean FBS from 205.30 (58.63) at baseline to 184.00 (67.58) at 6-month follow-up (t3), while the comparison group showed a mean FBS from 176.41 (40.40) at baseline to 168.91 (49.47) at 6-month follow-up. In terms of within-group change, only the CETA group showed a statistically significant improvement with a mean difference of −25.43 (−49.79 to −1.07) at the overall follow-up. HbA1C levels showed no change for either group from t1 to t3 (Table 2).

The results of the overall adjusted difference between the CETA and comparison groups (midline-endline) based on GEE showed that the CETA group exhibited a greater reduction in mental health symptoms compared to the comparison group (mean difference = −3.06, 95%CI: −5.19 to −0.93, p = 0.05). Other secondary outcomes were also improved, with no significant differences by condition.

## Discussion

The CETA intervention has been previously shown to be effective for reducing symptoms of depression, anxiety and posttraumatic stress and reducing substance use behaviors among populations living in humanitarian emergencies. This study provides preliminary evidence for the use of CETA to improve NCD medication adherence regardless of the participant’s mental health symptom score. CETA includes elements that are frequently used to improve medication adherence, including motivational interviewing, behavioral activation, cognitive coping and problem solving, which is consistent with other research; for example, Spoelstra et al. (2015) found effect sizes of 0.19 to 0.35 in four cohort studies combining motivational interviewing with cognitive behavior for enhancing medication adherence. The moderate effect sizes in our study (Cohen’s d = 0.38 for t2 and 0.23 for t3) were similar to those reported in the Spoelstra cohort studies. Other studies have also demonstrated that diabetic and hypertensive patients who received cognitive behavioral therapy showed better self-reported medication adherence than those in comparison conditions, supporting the potential benefit of behavioral interventions in chronic disease management (Cummings et al., 2019; Li et al., 2021; Abbas et al., 2023). CETA’s flexible, transdiagnostic design allows for ongoing refinement based on emerging evidence and implementation learnings across diverse settings. This trial extends CETA’s evidence base to medication adherence as a behavioral health outcome, demonstrating the adaptability of the approach to address non-traditional mental health targets while maintaining effectiveness for mental health symptoms.

The statistically significant intervention effect was found for self-reported medication adherence but not for the other method used to assess adherence: pill count. Measuring medication adherence is a challenging endeavor as no single method is universally accepted and different measures may produce different results (Stewart et al., 2014). In this study, eligibility was determined by pill count <70%, yet most participants reported “adequate adherence” on MARS-5 at baseline, indicating a substantial inconsistency between the two measures that warrants careful interpretation of the main findings. This discrepancy may also partly reflect differences in MARS-5 cut-offs used across studies, as some classify any MARS-5 score below 25 as poor adherence, whereas we used a three-category classification to align with our pill count categories, which may have contributed to higher baseline MARS-5 adherence estimates. Self-reported adherence may also be affected by social desirability bias and differences in comprehension or interpretation of questionnaire items, which may lead participants to over-report adherence.

In addition, there were challenges in operationalizing pill count measurement. Some patients took medications not only from the clinic but also from district hospitals, making dispensary information sometimes incomplete. Patients sometimes combined their pills from prior visits with the new ones and some patients also combined their pills with their family members with the same disease, making it difficult for data collectors to accurately count the pills. Given that pill count was used as an eligibility criterion, these limitations may have affected the precision of adherence classification at baseline. These challenges also highlight the need to supplement pill count records with other methods, such as self-reported adherence, to effectively identify patients who need adherence support.

Differences between pill counts, which identified some patients as poor adherers, and self-reports, which indicated adequate adherence, may reflect discrepancies between patients’ perceived intentions and actual behaviors, or limitations in the accuracy of pill count as an objective measure. Although self-reports may be subject to bias, they provide valuable insights into patients’ attitudes and motivation. The observed improvements in self-reported adherence suggest that participants may have experienced an increase in motivation to adhere to treatment. Importantly, any social desirability bias would be expected to operate similarly across study arms, so the observed between-group improvement over time in MARS-5 still supports an intervention effect. This shift in motivation is a key component of behavior change and indicates that the CETA program may have had a meaningful impact, even in the absence of statistically significant changes in pill count, which showed mean-level trends in the same direction as MARS-5.

As expected, the CETA program contributed to a meaningful reduction in mental health symptoms among those with elevated symptoms at baseline, a finding consistent with previous studies using CETA among Burmese refugees in Thailand and across different populations (Bolton et al., 2014; Bonilla-Escobar et al., 2018; Murray et al., 2020). The other clinical outcomes (blood pressure and blood sugar levels) were not found to be more greatly improved through CETA participation. This lack of effect may in part be a result of effective management of blood pressure and blood sugar levels requiring lifestyle behavioral changes, which are constrained for displaced people because of structural determinants such as a lack of healthy food availability and safe spaces for mobility; it was difficult for study participants to gain full control over their blood pressure and blood sugar levels even if they were motivated to do so.

In terms of limitations, the study had a relatively short follow-up period (6 months), which limits our ability to assess the long-term effectiveness of CETA on medication adherence. There may also be social desirability bias in the self-reported medication adherence, as participants might have been inclined to provide answers that reflect positively on themselves. The inconsistencies in baseline comparisons of the two adherence measures do need to be acknowledged, as it may have resulted in some misclassification of participants as study eligible. While the comparison of the CETA participants to the available MHPSS services showed impacts in the expected direction for both medication adherence outcomes, the impact of misclassification could have limited our ability to observe a statistically significant impact on pill-count and may have attenuated our results for the MARS-5 measure. Additionally, there were structural challenges, such as poor access to nutritious food and a lack of spaces for physical activity, that could affect the impact on the HT and DM physical health outcomes independent of medication adherence. Future research should also focus on dietary modification and lifestyle changes as integral components of chronic disease management to improve clinical outcomes.

In conclusion, our findings suggest that a mental health intervention, such as CETA, which incorporates behavioral change components, can be used to address health behavior issues such as medication adherence even in the absence of significant mental health concerns, while also addressing mental health concerns for people with poor medication and mental health symptoms. Humanitarian programs should consider integrating evidence-based mental health interventions like CETA into existing NCD care models to enhance both mental health and behavioral outcomes for patients.

## Data Availability

The datasets generated and/or analyzed during the current study are not publicly available due to privacy or confidentiality agreements but are available from the corresponding author on reasonable request.

## Long descriptions

### Figure 1. Long description

At the top, 1,712 patients in the I R C chronic database are screened. To the right, 1,062 are excluded for reasons including being busy (238), dead (139), not found (417), pilot (15), and other reasons (253). Downward, 650 are assessed for eligibility. To the right, 426 are excluded for not meeting criteria (358), declining participation (39), or other reasons (29). Downward, 224 are randomized. This splits into two branches: left, 112 allocated to C E T A, with 106 receiving intervention; right, 112 allocated to comparison, receiving routine services and 42 later receiving voluntary C E T A. In follow-up, left branch shows 6 discontinued C E T A and 10 lost to follow-up; right branch shows 7 withdrew and 9 lost to follow-up. In analysis, left branch includes all 112, with 105 analyzed at t2 and t3; right branch includes all 112, with 105 at t2 and 107 at t3.

Navigate back to Figure 1..

### Table 1. Long description

From the top, the table lists factors in the first column, with subsequent columns for intervention group (n equals 112, number and percent), comparison group (n equals 112, number and percent), and p-value. For age, intervention group mean is 56.08 years (SD 12.86), median 57.5 (range 24 to 88); comparison group mean is 55.27 (SD 11.68), median 56 (range 26 to 88), p-value 0.621. For sex, intervention group has 36 males (32.14 percent), 76 females (67.86 percent); comparison group has 29 males (25.89 percent), 83 females (74.11 percent), p-value 0.303. Marital status: intervention group 43 single/separated/divorced (38.39 percent), 69 married (61.61 percent); comparison group 31 single/separated/divorced (27.68 percent), 81 married (72.32 percent), p-value 0.088. Ethnic group: Karen 79 (70.54 percent) in both groups, Bamar 10 (8.93 percent) intervention, 3 (2.68 percent) comparison, Kachin 2 (1.79 percent) intervention, 1 (0.89 percent) comparison, Mon 2 (1.79 percent) intervention, 3 (2.68 percent) comparison, Others 19 (16.96 percent) intervention, 26 (23.21 percent) comparison, p-value 0.231. Religion: Christianity 31 (27.68 percent) intervention, 34 (30.36 percent) comparison; Buddhism 46 (41.07 percent) intervention, 36 (32.14 percent) comparison; Islam 35 (31.25 percent) intervention, 42 (37.50 percent) comparison, p-value 0.369. Duration of stay at shelter: less than 10 years, 6 (5.36 percent) intervention, 7 (6.25 percent) comparison; 10 to 19 years, 67 (59.82 percent) intervention, 76 (67.86 percent) comparison; 20 or more years, 39 (34.82 percent) intervention, 29 (25.89 percent) comparison; mean 18.32 (SD 6.87) intervention, 16.58 (SD 5.95) comparison; median 17 (range 1 to 35) intervention, 16 (range 1 to 32) comparison, p-value 0.348. Number of household members: 1 to 3, 26 (23.21 percent) intervention, 21 (18.75 percent) comparison; 4 to 6, 54 (48.21 percent) intervention, 58 (51.79 percent) comparison; more than 6, 32 (28.57 percent) intervention, 33 (29.46 percent) comparison, p-value 0.708. Category of disease: hypertension 69 (61.61 percent) intervention, 74 (66.07 percent) comparison; diabetes 22 (19.64 percent) intervention, 18 (16.07 percent) comparison; D M and H T comorbidity 21 (18.75 percent) intervention, 20 (17.86 percent) comparison, p-value 0.741. Statistical tests used are t-test, Chi-square, and Fisher’s exact test as indicated by superscripts.

Navigate back to Table 1..

### Table 2. Long description

From top to bottom, the table lists outcome domains: Pill count adherence, M A R S-5 adherence, mental health symptoms, blood pressure, fasting blood sugar, and H b A 1 C. For each, columns are grouped by assessment time: t1 baseline, t2 3-month, t3 6-month. Each timepoint is subdivided into C E T A and comparison arms, with number and percent or mean (standard deviation) shown. Pill count: At baseline, both groups had low good adherence (C E T A 0 percent, comparison 0 percent). At 3 months, good adherence rose to 69.52 percent in C E T A and 61.90 percent in comparison. At 6 months, good adherence was 72.38 percent in C E T A and 68.22 percent in comparison. Mean pill count adherence increased from 22.41 (24.72) at baseline to 77.05 (33.34) at 6 months in C E T A. M A R S-5: Adequate adherence increased from 93.75 percent at baseline to 99.05 percent at 6 months in C E T A. Mean M A R S-5 score rose from 20.83 (3.57) to 24.14 (1.68) in C E T A. Mental health: At baseline, all had elevated symptoms. At 3 months, low symptoms were seen in 83.33 percent of C E T A and 48.65 percent of comparison. At 6 months, low symptoms were 72.22 percent in C E T A and 62.16 percent in comparison. Mean mental health score dropped from 13.02 (7.35) to 3.83 (4.97) in C E T A. Blood pressure: At baseline, all had high S B P or D B P. At 6 months, normal blood pressure was seen in 69.77 percent of C E T A and 57.45 percent of comparison. Mean systolic dropped from 141.81 (16.06) to 124.41 (19.08) in C E T A; mean diastolic from 91.09 (8.11) to 80.04 (11.06). Fasting blood sugar: At baseline, all had high values. At 6 months, normal fasting blood sugar was seen in 22.58 percent of C E T A and 17.39 percent of comparison. Mean fasting blood sugar decreased from 205.30 (58.63) to 184.00 (67.58) in C E T A. H b A 1 C: At baseline, all had high values. At 6 months, normal H b A 1 C was seen in 3.57 percent of C E T A and 6.90 percent of comparison. Mean H b A 1 C decreased from 9.55 (1.94) to 9.35 (2.04) in C E T A. Significant improvements are marked by asterisks for p-values less than 0.05, 0.01, or 0.001.

Navigate back to Table 2..

### Table 3. Long description

The table has seven columns. The first column lists factors: Medication nonadherence, with sub-rows for Pill counts and MARS-5. The next four columns show adjusted post-intervention differences between CETA and comparison groups at t2 and t3, each with mean difference and p-value. The last two columns show overall difference between groups across t2 and t3 by G E E, with mean difference and p-value. For Pill counts, mean differences are 4.77 (negative 4.54 to 14.08) at t2, 5.71 (negative 4.03 to 15.46) at t3, and 4.96 (negative 2.26 to 12.19) overall, with p-values 0.314, 0.250, and 0.178, none significant. For MARS-5, mean differences are 0.73 (0.14 to 1.33) at t2, 0.39 (negative 0.12 to 0.91) at t3, and 0.55 (0.07 to 1.02) overall, with p-values 0.015, 0.137, and 0.023. Significant improvement is observed for MARS-5 at t2 and overall, but not for Pill counts.

Navigate back to Table 3..

### Table 4. Long description

The table contains seven columns. The first column lists primary healthcare, health status, and medication adherence factors, with subcategories indented beneath. The next four columns show adjusted post-intervention differences between comparison and CETA groups by A N C O V A, split into midline and endline, each with mean difference and p-value. The last two columns show overall difference between groups across midline and endline by G E E, with mean difference and p-value. Under primary healthcare, mental health symptoms show a midline mean difference of minus 3.53 (95 percent confidence interval minus 5.52 to minus 1.55), p-value 0.001; endline mean difference minus 2.58 (minus 5.63 to 0.45), p-value 0.094; overall mean difference minus 3.06 (minus 5.19 to minus 0.93), p-value 0.005. For hypertension, systolic blood pressure at midline is minus 0.80 (minus 8.61 to 7.00), p-value 0.838; endline minus 2.72 (minus 10.86 to 5.42), p-value 0.509; overall minus 1.76 (minus 8.02 to 4.49), p-value 0.581. Diastolic blood pressure at midline is 3.46 (minus 2.79 to 9.73), p-value 0.274; endline 0.84 (minus 3.56 to 5.25), p-value 0.704; overall 2.15 (minus 1.87 to 6.19), p-value 0.295. Fasting blood plasma at midline is minus 16.47 (minus 54.50 to 21.54), p-value 0.388; endline minus 1.38 (minus 33.06 to 30.28), p-value 0.930; overall minus 9.05 (minus 34.06 to 15.95), p-value 0.478. For H b A 1 C, only endline data is reported: mean difference 0.59 (0.34 to 0.84), p-value less than 0.001. N a is listed for missing values. All differences are adjusted to baseline.

Navigate back to Table 4..
