# Supplementary material for: Effectiveness of psychotherapy in improving medication adherence for hypertension and diabetes mellitus among displaced Myanmar patients in Thailand: A randomized control trial
Source: Glob Ment Health (Camb). 2026 May 26;13:e155. doi: 10.1017/gmh.2026.10225 (PMC13375837; doi:10.1017/gmh.2026.10225)
Supplement: Peer review history

# Reviewer report: Review: Effectiveness of psychotherapy in improving medication adherence for hypertension and diabetes mellitus among displaced Myanmar patients in Thailand: A randomized control trial — R0/PR2

**DOI:** 10.1017/gmh.2026.10225.pr2

The authors explored whether an intervention designed to address psychiatric complaints found in a refugee population would beneficially impact medication compliance for hypertension and type II diabetes mellitus, the latter referred to as “noncommunicable diseases (NCD)”. The intensity of this intervention (i.e., “Common Elements Treatment Approach [CETA]”) was titrated for reported comorbid behavioral symptoms. Follow-up data were collected at three and six months after administration of the intervention. Medication compliance was assessed with pill counts and self-reported 5-item Medication Adherence Rating Scale. Rationale, study design and statistical methodology were excellent.

The authors are to be commended for exploring/developing a therapeutic behavioral intervention in this large and heterogeneous displaced refugee population. References and citations are provided that address effectiveness and (presumably) efficiency of the specific CETA intervention for primarily behavioral outcomes (e.g., Murray et al., 2014; Bolton et al., 2014; Murray et al., 2020), as well as citations supporting cognitive psychotherapeutic interventions improving treatment adherence for NCD. For readers unfamiliar with specific components of CETA, the authors could devote additional sentences to conveying what transpires between “therapist and patient” during the actual intervention (I suspect that in addition to individualizing the intensity in terms of titrating “number of sessions”, there is also variability with respect to session “content” across patients.) Is the CETA intervention manual-driven and do supervisory sessions collect data to show that therapists administering the intervention adhere to guidelines, in an effort to minimize variability across therapists.

The results show modest superiority of CETA over the “treatment as usual” comparison condition, which neither distracts nor diminishes my enthusiasm for the trial and dissemination of its results. I am wondering if CETA is still a work in-progress with data-driven iterative changes made to the intervention itself to improve its “effectiveness (i.e., treatment outcome)” and “efficiency” of its delivery.

# Reviewer report: Review: Effectiveness of psychotherapy in improving medication adherence for hypertension and diabetes mellitus among displaced Myanmar patients in Thailand: A randomized control trial — R0/PR3

**DOI:** 10.1017/gmh.2026.10225.pr3

**Authors:** Chuo Yew Ting

**Affiliations:** Sarawak State Hlth Dept

The design of the study is methodologically rigorous and well executed. However, the manuscript lacks a clear theoretical contribution, as no conceptual framework grounded in psychological or behavioral theory is presented. Without such a framework, it is difficult to understand the underlying mechanisms through which the Common Elements Treatment Approach (CETA) influences medication adherence. Furthermore, because CETA is a flexible, non-standardized intervention and participants received varying durations and components of therapy, the generalizability of the findings is limited. The study would be strengthened by the inclusion of a conceptual model illustrating how CETA operates within established psychological and behavioral theories, such as the Transtheoretical Model, Health Belief Model, or Theory of Planned Behavior.

# Reviewer report: Review: Effectiveness of psychotherapy in improving medication adherence for hypertension and diabetes mellitus among displaced Myanmar patients in Thailand: A randomized control trial — R0/PR4

**DOI:** 10.1017/gmh.2026.10225.pr4

**Authors:** Miriam Arroyo

Title: Effectiveness of psychotherapy in improving medication adherence for hypertension and diabetes mellitus among displaced Myanmar patients in Thailand: A randomized control trial

Manuscript ID: GMH-2025-0316

SUMMARY OF THE MANUSCRIPT

The manuscript addresses a timely and relevant topic at the intersection of mental health, chronic disease management, and humanitarian health systems. Evaluating the Common Elements Treatment Approach (CETA) through a randomized controlled trial (RCT) to indirectly improve medication adherence among displaced adults with hypertension and diabetes is conceptually appropriate and adds valuable evidence to the global mental health literature. However, despite these strengths, the manuscript requires important refinements. In its current form, the study would benefit from additional methodological clarification, deeper theoretical grounding, and a more interpretation of its findings—especially regarding the implications for the specific population under study.

SPECIFIC COMMENTS

Introduction. The introduction would benefit from a more explicit theoretical framework explaining how a mental health intervention such as CETA might improve medication adherence even among individuals without mental health symptoms (Eg. planning, problem-solving, routine formation, regulation of stress and cognitive load in humanitarian settings). Strengthening the conceptual grounding will clarify the rationale for the trial and interpretation of results.

The theoretical grounding in the introduction would benefit from slightly clearer organization. The paragraph describing the history and demographics of the displaced Myanmar population provides valuable context (Page 2, 14-23); however, placing it earlier in a background section might help maintain the logical flow of the argument. Once repositioned, the authors could strengthen the narrative by more explicitly connecting this contextual information to the specific vulnerabilities faced by camp residents—such as exposure to trauma, chronic stress, limited autonomy, and resource constraints—which may heighten both mental health risks and difficulties with long-term medication adherence.

Methods. A methodological concern is the significant inconsistency between the two measures of medication adherence:

• Eligibility for the trial was determined by pill count <70%.

• Yet 93.75% of participants reported “adequate adherence” on MARS-5 at baseline, which contradicts the objective eligibility criterion (Page 6, 40-42).

This discrepancy raises concerns regarding interpretation of the main finding, since the statistically significant improvement is observed only in MARS-5. The manuscript acknowledges challenges in pill count implementation, but this issue warrants a more comprehensive methodological discussion, including:

• Possible biases in self-report (social desirability, cultural norms, comprehension).

• Structural, contextual, and logistical limitations that may compromise pill count validity.

• Implications of these inconsistencies for internal validity and interpretation of results.

• Whether the eligibility criterion may have introduced misclassification.

This must be expanded in the Discussion and Limitations sections.

The manuscript would benefit from a more explicit explanation of the nature of the interventions delivered in each arm of the trial. Without a clearer differentiation between the active therapeutic elements of CETA and those embedded in routine psychosocial care, it becomes difficult to interpret the source of the observed effects. Providing this distinction would substantially strengthen the interpretation and internal validity of the study’s conclusions.

The procedure for randomization needs clearer detail:

• How was allocation concealment ensured using “opaque sealed paper”?

• Who prepared the randomization sequence and envelopes?

• Was the sequence tamper-proof?

Clearer description is needed to assess risk of allocation bias.

Participants. A clearer description of how mental health status was assessed at enrollment would strengthen the manuscript. Given the high vulnerability of displaced populations, it would be useful to clarify why certain mental health conditions (depression, anxiety, and PTSD) were selected for symptom evaluation and how the severity of mental health problems was defined and assessed for exclusion purposes (Page 3, 32-33)-

Relatedly, the manuscript notes that the number of CETA sessions was determined by the presence of “mental health situations,” but this term requires further clarification. It would be helpful to specify how these situations were identified and how they were differentiated from diagnosable mental disorders.

Providing information on the validity and reliability of the structured instruments used—particularly within this cultural and linguistic context—would enhance confidence in the study’s measurement approach.

Statistical Methods and Reporting. Choice of ANCOVA + GEE. The statistical rationale should be strengthened. Why were mixed-effects models not used, given their standard application in RCTs with repeated measures?

Results. The manuscript would benefit from applying statistical tests that align with the level of measurement of each variable when comparing baseline characteristics between trial arms (Table 1, p. 6, lines 6–20). Clarifying which tests were used—such as chi-square or Fisher’s exact tests for categorical variables, t-tests or non-parametric equivalents for continuous variables—would improve transparency and ensure that the reported comparisons accurately reflect the underlying data distributions.

Discussion. The conclusion that “CETA improves medication adherence” should be presented more cautiously. Several factors limit the strength of this inference, including:

• Inconsistency between adherence measures.

• Small effect size on MARS-5.

• Lack of effect on pill count.

• Short follow-up duration (6 months).

The Discussion should include a more nuanced interpretation of the magnitude and clinical relevance of the effects.

In particular, the manuscript should more clearly articulate the mechanisms through which CETA—relative to routine psychosocial support—could plausibly influence adherence behavior. As implemented in this trial, CETA does not include components specifically designed to change adherence behavior, and this should be explicitly acknowledged. If the authors believe CETA may improve adherence indirectly through mental health pathways, the manuscript should discuss whether mental health treatment alone is likely to be sufficient, or whether adherence-specific strategies would need to be integrated into real-world chronic disease programs.

As implemented in this trial, CETA does not include components specifically designed to change adherence behavior, and this should be explicitly acknowledged. If the authors believe CETA may improve adherence indirectly through mental health pathways, the manuscript should discuss whether mental health treatment alone is likely to be sufficient, or whether adherence-specific strategies would need to be integrated into real-world chronic disease programs.

Overall, while the topic is important and the design appropriate, the manuscript would be strengthened by clarifying these conceptual and methodological issues and by providing a more nuanced discussion of the implications—or lack thereof—of the intervention effects on medication adherence.

# Editor report: Recommendation: Effectiveness of psychotherapy in improving medication adherence for hypertension and diabetes mellitus among displaced Myanmar patients in Thailand: A randomized control trial — R0/PR5

**DOI:** 10.1017/gmh.2026.10225.pr5

**Affiliations:** Pravara Institute of Medical Sciences, India

All three reviewers have raised major concerns related to the theoretical underpinnings of this work. How the intervention is likely to have an effect on the outcome needs to be clearly articulated. There are several other concerns that the reviewers have raised, and they need to be addressed by the authors.

# Editor report: Decision: Effectiveness of psychotherapy in improving medication adherence for hypertension and diabetes mellitus among displaced Myanmar patients in Thailand: A randomized control trial — R0/PR6

**DOI:** 10.1017/gmh.2026.10225.pr6

**Affiliations:** London School of Hygiene & Tropical Medicine, United Kingdom of Great Britain and Northern Ireland

No accompanying comment.

# Editor report: Recommendation: Effectiveness of psychotherapy in improving medication adherence for hypertension and diabetes mellitus among displaced Myanmar patients in Thailand: A randomized control trial — R1/PR8

**DOI:** 10.1017/gmh.2026.10225.pr8

**Affiliations:** Pravara Institute of Medical Sciences, India

No accompanying comment.

# Editor report: Decision: Effectiveness of psychotherapy in improving medication adherence for hypertension and diabetes mellitus among displaced Myanmar patients in Thailand: A randomized control trial — R1/PR9

**DOI:** 10.1017/gmh.2026.10225.pr9

**Affiliations:** London School of Hygiene & Tropical Medicine, United Kingdom of Great Britain and Northern Ireland

No accompanying comment.
